# The American Hellebore—V. Viride

**Published:** 1854-03

**Authors:** 


### ART. II.— *The American Hellebore— V. Viride.*

By Rusticus.

Messrs. Editors, — It may be that you have heard enough of V. viride. Perhaps you have come to think its name appropriate — that it is not only *hellebore*, but a bore in every other sense of the word.

Be not offended, if I, too, bring up this shade of antiquity to your notice. It is an ancient and approved remedy, and though it, like many other drugs, has become a *caput mortuum*, an useless waif upon the stream of progress, cast ashore ages ago by the ebbing waters, yet when it again appears, like some weird ghost upon the stage, it should meet with becoming reverence.

Drugs have an individuality. Resident in some of them is a certain force, which attracts some minds, while it repels others. Thus between the hellebore and Dr. Norwood, of Cokesbury, S. C., there is an elective affinity. Dr. N. was made for hellebore, and it is becoming evident that hellebore was made for Dr. N. More than this. *It bids fair to make him.* The hills of Vermont have yielded up their simples; the veratrum is piled by hundreds weight in New York drug-stores, and manufactured into tinctures, put up in four ounce bottles, with the name and seal of Norwood on them — none other genuine — for sale at the principal drug-stores in all the states of the Union, where circulars may be had gratuitously. The profession may rely on the purity of the article if they purchase this brand, but — beware of counterfeits! Recommended by the faculty in coughs, colds, consumption, and liver complaints; pneumonia, typhoid fever, and the rest of antipodal diseases.

A medicine thus offered to our notice, demands a careful consideration. Such, Messrs. Editors, do I propose to give it.

I like to begin at the beginning. It happened a very long time ago, that the daughters of King Praetus were ill, and daily grew more thin and melancholy. Some sad humor (so the story runs) infected their brains, and they drew nigh to the very sill of the door of death. A certain shepherd-physician, named Melampus, cured these frail sisters, by giving them the milk of goats which were fed on hellebore. Thus it happens that some old books call hellebore, Melampodium, in honor of him who cured the princesses.

Having thus gained the favor and sanction of a royal family, hellebore had free course thereafter. But the first distinct notice of its medical properties, I find in the Aphorisms of Hippocrates:

"Sectio IV. Aphorismus 13. Ad elleboros, qui non facilè sursum purgantur, iis ante potionem corpora præhumectanda, copiosiore alimento, et quiete."

"14. Ubi biberit quis elleborum, ad motiones quidem corporum magis ducito: ad somnos verò, et quietem, minus."

I omit the remainder of this, and the succeeding chunk of wisdom, because here, as in many other places, Hippocrates repeats himself.

"16. Elleborus periculosus est sanas carnes habentibus: convulsionem enim inducit."

That is, hellebore will give a healthy man fits. I appeal to this as an evidence of the progress of medical art. In the days of Hippocrates it caused fits; in the days of Dr. Norwood it cures them. O! mighty march of Intellect! which through the ages grows in wisdom and in stature! etc., etc., &c., &c.

That we may not be mistaken as to the true views of the Coan Sage, I quote another aphorism:

"Convulsio ab ellebori, lethale."

"A fit from hellebore—a dead man!" Pithy, that!

For many centuries hellebore was thought to purge the brain. It was considered exceeding good in distempers of the mind. Hence we find not unfrequent allusions to it in the satirical poets, and especially in the satires of Horace.

"Danda est ellebori multò pars maxima avaris:

"Nescio an Anticyram ratio illis destinet omnem."—Sat. Lib., 11, 3.

Here he recommends it to the avaricious, but doubts whether all Anticyras would furnish enough to bring them to their senses. The island of Anticyras furnished the principal supply of the root. The modern Anticyras is in Vermont, whither Dr. Norwood bends his steps from the far Carolinas, in search of that which may purge, not only the modern brain, but any other organ afflicted by the offspring of Pandora's box.

I have often expressed to you my conviction, that new things happen only once in a great while; that most of the fancied discoveries of modern days are but the reproduction of old time theories. What is new to us, is not necessarily new to the rest of the world. Our fancies, and our interest, lead us, not unfrequently, into extravagances. When, as in the instance of hellebore, we find that a remedy has previously undergone the test of ages, has been found wanting, and has disappeared from the scene of action, we should hesitate how we mount it as a hobby.

And now, Messrs. Editors, I wish to lay down a proposition, a kind of "stand-point of view," as the redundant men of the west have the phrase. No scientific man—no man decently versed in the laws of morbid action—

would claim for a remedy, equal virtue, and specific curative effect, in two such diseases as pneumonia and typhoid fever.

We should not suffer our good nature to lead us to endorse theories and medicines of doubtful value. The variety of diseases for which the V. viride has been prescribed; the unfailing certainty with which it cures them all, (if we believe its advocates,) and more than this, the fact that it is claimed to cut short diseases which depend upon blood poisons, should lead us at once to the conclusion, that the observers of the action of this drug are not trustworthy, because deficient in knowledge.

If the drug acts by lowering the heart's action, how is it that it succeeds not only in sthenic diseases, but in typhoid lesions, where the action of the heart is already enfeebled? If it exerts such a wonderful influence over excited conditions of the nervous system, such as convulsions, how is it that it is equally applicable in depressed nervous conditions?

It strikes me, Messrs. Editors, that the flowers of fancy bloom upon the stalk of veratrum viride; and that, as in the Augustan age, it works wonders with the brains of those who dabble in it. In its various forms of white, black, and green, it has been the subject of more absurd hypotheses than any drug in the whole catalogue of Wood and Bache. The ancients subjected it to all manner of analyses, and destructive distillations; they dissolved it in acids, and burned it in retorts; and at last gave it up as an uncertain and dangerous narcotic poison, which sometimes physicked and sometimes puked; which cured fits, and caused fits; until, at last, the whole value of their knowledge might be summed up in the short and spicy aphorism of Hippocrates: "Convulsio ab ellebore—*lethale*." I doubt whether that does not tell, as well, the story of modern knowledge.

The old writers divided the class purgatives into emetics, and physics. Thus Hippocrates speaks of "sursum purgante," and of "purgante deorsum." Mons. Bolduc (Phil. Trans. n. 278, p. 1099) makes some nice distinctions as to the resinous and woody parts of the hellebore, and recommends that it be prepared in an aqueous menstruum, to avoid its dangerous effects. Perhaps your readers will be interested in a learned account of sundry chemical analyses of the hellebore by Mons. Bolduc. "He said, that having put it in a Retort in a Reverberatory fire, he at first drew off an Acid Spirit, next an Oily Acid Spirit, thirdly a violent *Alkali* Spirit came over mixt with Oil of Tartar, and lastly a fœtid Oil."

I submit that any drug, containing such mysteriously horrible ingredients, should be never administered except in accordance with the following tables, invented to govern its use by Dr. W. Cockburn, in March, 1704, Old Style:

"By a further Disquisition into this Matter, we find that the Doses must not only be greater where the Thickness of Blood is greater; but that they must be encreas'd in a duplicate Proportion of their Viscidity. This is evident by the Tables in *Cassia*, viz., 9: 8℥ :: 4: 3℥, 1℈, 13⅔ gr., and therefore *alternando* 9: 4:: 8℥ : 3℥, 1℈, 13⅔. Therefore the Doses are as the Squares of the Constitutions. So likewise 9: 8℥ :: 16: 14℥, 13⅔ gr. and *alternando* 9: 16:: 8℥ : 14℥, 13⅓ gr. N. e. the Doses are as the Squares of the Constitutions."

Hence, O Doctor Norwood! you have the following:

"*A Problem.*" { "*The Quantity of Blood in any Person being given, together with the ordinary and extraordinary Effect of a Dose of a Purging Medicine, the Change of that Person's Constitution, and the Nature of that Change may be determined.*"—*Q. E. D.*

——— + Roads, February 4, 1854.

